# Supplementary material for: Extended DNA threading through a dual-engine motor module of the activating signal co-integrator 1 complex
Source: Nat Commun. 2023 Apr 5;14:1886. doi: 10.1038/s41467-023-37528-3 (PMC10076317; doi:10.1038/s41467-023-37528-3)
Supplement: Supplementary file 1 — Supplementary information [file 41467_2023_37528_MOESM1_ESM.pdf]

Supplementary Information

Extended DNA threading through a dual-engine motor module of the activating signal co-integrator 1 complex

Junqiao Jia[1,§], Tarek Hilal[1,2], Katherine E. Bohnsack[3], Aleksandar Chernev[4], Ning Tsao[5], Juliane Bethmann[4,6], Aruna Arumugam[1], Lane Parmely[5], Nicole Holton[1], Bernhard Loll[1], Nima Mosammaparast[5], Markus T. Bohnsack[3,7,8], Henning Urlaub[4,6], Markus C. Wahl[1,9,*]

[1] Freie Universität Berlin, Institute of Chemistry and Biochemistry, Laboratory of Structural Biochemistry, Takustr. 6, D-14195 Berlin, Germany

[2] Freie Universität Berlin, Institute of Chemistry and Biochemistry, Research Center of Electron Microscopy, Fabeckstr. 36a, D-14195 Berlin, Germany

[3] Universitätsmedizin Göttingen, Department of Molecular Biology, Humboldallee 23, D-37073 Göttingen, Germany

[4] Max-Planck-Institut für Multidisziplinäre Naturwissenschaften, Bioanalytical Mass Spectrometry, Am Fassberg 11, D-37077 Göttingen, Germany

[5] Washington University School of Medicine, Department of Pathology & Immunology and Center for Genome Integrity, 660 S. Euclid Ave, St. Louis, MO 63110, USA

[6] Universitätsmedizin Göttingen, Institut für Klinische Chemie, Bioanalytik, Robert-Koch-Straße 40, D-35075 Göttingen, Germany

[7] Georg-August-Universität, Göttingen Center for Molecular Biosciences, Justus-von-Liebig-Weg 11, D-37077 Göttingen, Germany

[8] Max-Planck-Institut für Multidisziplinäre Naturwissenschaften, Am Fassberg 11, D-37077 Göttingen, Germany

[9] Helmholtz-Zentrum Berlin für Materialien und Energie, Macromolecular Crystallography, Albert-Einstein-Str. 15, D-12489 Berlin, Germany

[§] Present address: Harvard Medical School, Department of Cell Biology, 240 Longwood Avenue, Boston, MA 02115, USA

* Correspondence to: markus.wahl@fu-berlin.de

Supplementary Tables

Supplementary Table 1: CryoEM data collection, refinement and validation statistics.

**Data collection and processing**	
Microscope	FEI Titan Krios G3i
Voltage [keV]	300
Camera	Falcon 3EC
Magnification (nominal)	96,000x
Pixel size at detector [Å/pixel]	0.832
Total electron exposure [e^-/$Å^2$]	42
Exposure rate [e^-/pixel/s]	0.7
Frames collected during exposure	33
Defocus range [µm]	0.8 - 2
Automation software	EPU (version 2.14; Thermo Fisher Scientific)
Micrographs	
Collected	6269
Used	6024
Particle images	
Total extracted	2,819,378
Refined	1,590,881
Final	244,064
Point-group or helical symmetry parameters	C1
Resolution [Å]	
Global	3.4
$FSC_{0.143}$[a] (unmasked/masked)	3.8/3.4
Local resolution range [$Å^2$]	2.5 - 35
Map sharpening B factor/range [$Å^2$]	134.9
Map sharpening methods	local B-factor
Refinement software	
Package	PHENIX (version 1.20_4459)
Routine	real.space.refine
**Refinement**	
Model composition	
Non-hydrogen atoms	16,409
Protein residues (ASCC3/TRIP4)	256/1,783
Zn^{2+} ions	2
Model-Map scores	
CC[b] (mask)	0.79
CC (volume)	0.78
Average grouped B factors [$Å^2$]	
Overall	133
Protein (ASCC3/TRIP4)	146/131
Zn^{2+} ions	164
Rmsd[c] from ideal values	
Bond lengths [Å]	0.003
Bond angles [°]	0.717
**Validation**[d]	
MolProbity score	2.00
CaBLAM outliers [%]	2.7
Clashscore	15.3
Poor rotamers [%]	0.4
Cβ deviations	0
EMRinger score	0.63
Ramachandran plot	
Favored [%]	95.5
Allowed [%]	4.5
Outliers [%]	0.5

Ramachandran plot Z-score (rmsd)	
Overall	-0.35 (0.19)
Helices	0.81 (0.18)
Sheets	-0.45 (0.31)
Loops	-1.16 (0.21)
Data deposition	
Reconstruction	EMDB: EMD-15521
Coordinates	PDB: 8ALZ

[a] FSC, Fourier shell correlation
[b] CC, correlation coefficient
[c] Rmsd, root-mean-square deviation
[d] Using MolProbity[55]

Supplementary Table 2: Quantification of unwinding assays

	ASCC3^{HR}	ASCC3^{HR}-TRIP4	ASCC3^{HR}-TRIP4$^{1-230}$	ASCC3^{HR}-TRIP4$^{403-581}$	ASCC3^{HR,D1453A}	ASCC3^{HR,D1453A}-TRIP4	ASCC3^{HR}-ALKBH3
**Substrate**	DNA minus trap						
A_{fast} [(a)]	0.309	0.497	0.279	0.212	0.393	0.402	
A_{slow} [(a)]	0.691	0.503	0.721	0.788	0.607	0.598	
k_{fast} (s^{-1}) [(b)]	0.031	0.061	0.038	0.045	0.013	0.028	
k_{slow} (s^{-1}) [(b)]	0.005	0.010	0.005	0.004	0.002	0.004	
$k_{uaw}{}^{a}$(s^{-1}) [(c)]	0.024	0.054	0.030	0.035	0.011	0.024	
R^2 [(d)]	0.999	0.998	0.998	0.998	0.995	0.996	
**Substrate**	DNA plus trap						
A_{fast} [(a)]	0.294	0.495					0.295
A_{slow} [(a)]	0.706	0.505					0.705
k_{fast} (s^{-1}) [(b)]	0.027	0.067					0.024
k_{slow} (s^{-1}) [(b)]	0.004	0.006					0.004
$k_{uaw}{}^{a}$(s^{-1}) [(c)]	0.021	0.062					0.018
R^2 [(d)]	0.999	0.998					0.998
**Substrate**	RNA minus trap						
A_{fast} [(a)]	0.010	0.103					
A_{slow} [(a)]	0.990	0.897					
k_{fast} (s^{-1}) [(b)]	0.097	0.052					
k_{slow} (s^{-1}) [(b)]	0.0003	0.001					
$k_{uaw}{}^{a}$(s^{-1}) [(c)]	0.073	0.046					
R^2 [(d)]	0.999	0.999					

[a] A_{fast} and A_{slow} are the total unwinding amplitudes of the rapid and slow phases.
[b] k_{fast} and k_{slow} are the unwinding rate constant of the rapid and slow unwinding phases.
[d] k_{uaw} is the overall, amplitude-weighted unwinding rate constant, calculated as $k_{uaw} = (A_{fast} * k_{fast}{}^2 + A_{slow} * k_{slow}{}^2) / (A_{fast} * k_{fast} + A_{slow} * k_{slow})$.
[d] R^2 is the goodness of fit.

Supplementary Table 3: Synthetic gene sequence encoding TRIP4 (5’ to 3’)

TRIP4 synthetic gene sequence
ATGGCTGTCGCTGGTGCTGTTAGCGGAGAACCTTTGGTCCACTGGTGCACCCAGCAGCTGAGAAAGACCTTCG
GACTGGACGTGTCCGAAGAGATCATCCAGTACGTCCTGTCTATCGAGTCTGCCGAAGAAATCAGGGAATACGT
CACCGACCTGCTGCAGGGAAACGAAGGCAAGAAGGGACAGTTCATCGAGGAACTCATCACCAAGTGGCAGAAG
AACGACCAAGAGCTGATCTCTGACCCTCTGCAGCAGTGCTTCAAGAAGGACGAAATCCTGGACGGACAGAAGT
CTGGCGACCACTTGAAGCGCGGAAGAAAGAAGGGTCGCAACCGCCAAGAAGTCCCCGCCTTCACTGAACCTGA
CACCACCGCCGAAGTCAAGACCCCTTTCGACCTGGCCAAGGCTCAAGAGAACTCTAACTCCGTCAAGAAAAAG
ACCAAGTTCGTCAACCTGTACACCCGCGAAGGACAGGACAGGCTGGCTGTCTTATTGCCCGGAAGGCACCCTT
GCGACTGCCTGGGACAAAAGCACAAGCTGATCAACAACTGCCTGATCTGCGGAAGGATCGTCTGCGAACAAGA
AGGATCTGGACCCTGCCTCTTCTGCGGAACCCTTGTCTGCACCCACGAGGAACAGGACATCCTGCAGCGCGAC
TCTAACAAGTCTCAGAAGCTGCTGAAGAAACTGATGTCCGGCGTCGAGAACTCTGGAAAGGTGGACATCTCTA
CCAAGGACCTGTTGCCTCACCAAGAACTGAGGATCAAGAGCGGACTGGAAAAGGCCATCAAGCACAAGGACAA
GCTGCTCGAGTTCGACAGGACCTCTATCAGGCGCACCCAAGTCATCGACGACGAATCTGACTACTTCGCCTCT
GACTCTAACCAGTGGCTGTCTAAGCTGGAAAGGGAAACCCTGCAGAAGCGCGAAGAGGAACTGAGGGAACTGC
GCCACGCTTCTCGCCTGTCTAAGAAAGTCACCATCGACTTCGCCGGACGCAAGATCCTGGAAGAAGAAAACTC
TCTGGCCGAGTACCACTCTAGGCTGGACGAAACCATCCAGGCTATCGCCAACGGAACCCTGAACCAGCCTCTG
ACCAAGCTGGACAGGTCCTCTGAAGAACCCCTGGGAGTCCTGGTCAACCCCAACATGTACCAGTCTCCACCTC
AGTGGGTAGACCACACCGGTGCCGCTTCTCAGAAGAAGGCTTTCCGCTCCTCTGGATTCGGACTCGAGTTCAA
CTCTTTCCAGCACCAACTGCGCATCCAGGACCAAGAGTTCCAAGAGGGATTCGACGGTGGATGGTGCCTGTCT
GTCCACCAGCCTTGGGCTTCATTGCTCGTCAGGGGAATCAAGCGCGTCGAAGGACGCTCTTGGTACACCCCTC
ATCGTGGAAGGCTGTGGATCGCCGCTACCGCTAAGAAGCCCTCTCCACAAGAAGTCTCCGAGCTGCAGGCTAC
CTACAGGCTGTTGCGTGGAAAGGACGTCGAGTTCCCCAACGACTACCCCTCTGGATGCCTGCTGGGATGCGTT
GACCTGATCGACTGCCTCAGCCAGAAGCAGTTCAAAGAACAGTTCCCCGACATCTCCCAAGAGTCTGACTCTC
CCTTCGTGTTCATCTGCAAGAACCCTCAAGAGATGGTCGTGAAGTTCCCCATCAAGGGCAACCCTAAGATCTG
GAAGCTGGACTCTAAGATCCACCAGGGTGCCAAGAAAGGACTGATGAAGCAGAACAAGGCCGTCTAA

Supplementary Table 4: PCR primers (5’ to 3’)

Primer	Sequence
pETM11_syTRIP4_NcoI-F	GGTTTTCCATGGCTGTCGCTGGTGCTGTTAG
pETM11_syTRIP4_HindIII-R	GGTTTTAAGCTTTTAGACGGCCTTGTTCTGCTTCATC
piDK_syTRIP4_BamHI-F	GGTTTTGGATCCATGGCTGTCGCTGGTGCTGTTAG (pIDS)
pFL10his_syTRIP4_XbaI-R	GGTTTTTCTAGATTAGACGGCCTTGTTCTGCTTCATC (pIDS)
TRIP4_R80_HindIII	GGTTTTAAGCTTTTAAGAGATCAGCTCTTGGTCGT
TRIP4_R230_HindIII	GGTTTTAAGCTTTTACAGTTTCTTCAGCAGCTTC
TRIP4_F_152_NcoI	GGTTTTCCATGGCTCTGTACACCCGCGAAGGACA
TRIP4_F281_NcoI	GGTTTTCCATGGCTCAAGTCATCGACGACGAATCTG
TRIP4_R403_HindIII	GGTTTTAAGCTTTTAAGCCTTCTTCTGAGAAGCGG
TRIP4_F403_NcoI	GGTTTTCCATGGCTTTCCGCTCCTCTGGATTC
ALKBH3_FOR	ATATCCATGGCGATGGAGGAAAAAAGACGGCGAGCCCGAGTT
ALKBH3_REV	ATATGAATTCGGTCACCAGGGTGCCCCTCGAGGGTCTGGATAG
sgTRIP4-F	CACCGAAAGGTGGACATCTCTACCA
sgTRIP4-R	AAACTGGTAGAGATGTCCACCTTTC

Supplementary Figures

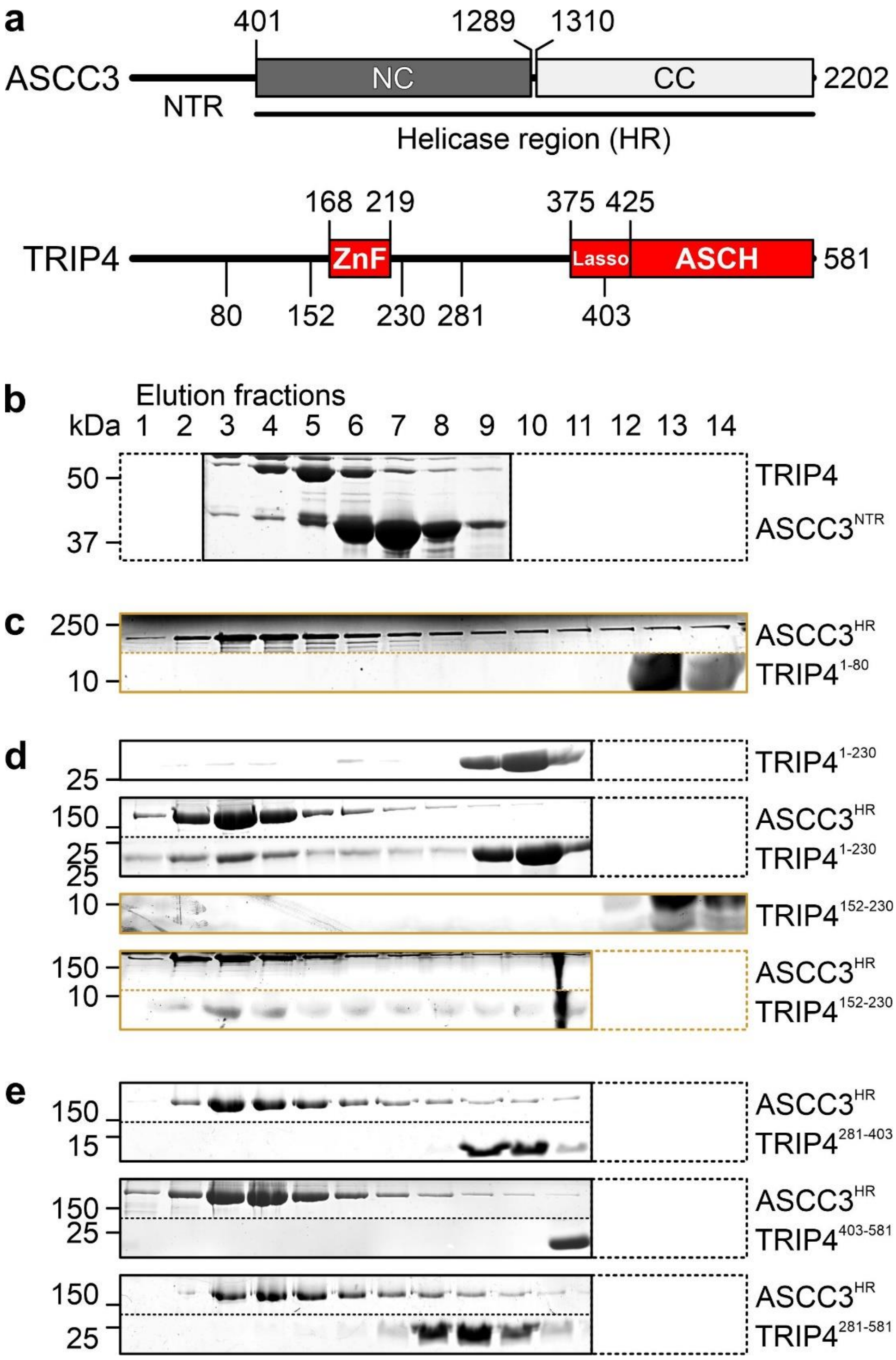

Supplementary Figure 1: ASCC3-TRIP4 interaction mapping.

a, Schemes of regions or domains in ASCC3 and TRIP4. Numbers above the schemes, region/domain borders; numbers below the TRIP4 scheme, borders of fragments employed. NTR, N-terminal region; NC/CC, N-terminal/C-terminal cassettes; HR, helicase region; ZnF,

zinc finger domain; Lasso, lasso peptide; ASCH, ASC-1 homology domain. **b-e**, SDS-PAGE analyses of analytical SEC elution fractions monitoring the interaction of ASCC3^{NTR} or ASCC3^{HR} with different regions of TRIP4. Throughout all panels, equivalent elution fractions are vertically aligned. Dashed regions indicate elution fractions that were not loaded on the gels. Molecular mass markers in kDa are shown on the left; protein bands are identified on the right. Black outlines, Coomassie stain; golden outlines, silver stain. For stably interacting TRIP4 fragments, analytical SEC runs of the isolated TRIP4 fragments are shown for comparison. For some analytical SEC runs, separate regions of the same gel were spliced together for display purposes (see Source Data file for uncropped gels). Dashed horizontal lines, splice lines. **b**, TRIP4 does not stably bind ASCC3^{NTR}. **c**, TRIP4$^{1-80}$ does not stably bind ASCC3^{HR}. **d**, TRIP4 fragments containing the ZnF domain (TRIP4$^{1-230}$, TRIP4$^{152-230}$) stably bind ASCC3^{HR}. **e**, C-terminal fragments of TRIP4 lacking the ZnF domain (TRIP4$^{281-403}$, TRIP4$^{403-581}$, TRIP4$^{281-581}$) do not stably bind ASCC3^{HR}. Experiments were repeated independently at least three times with similar results. Source data are provided as a Source Data file.

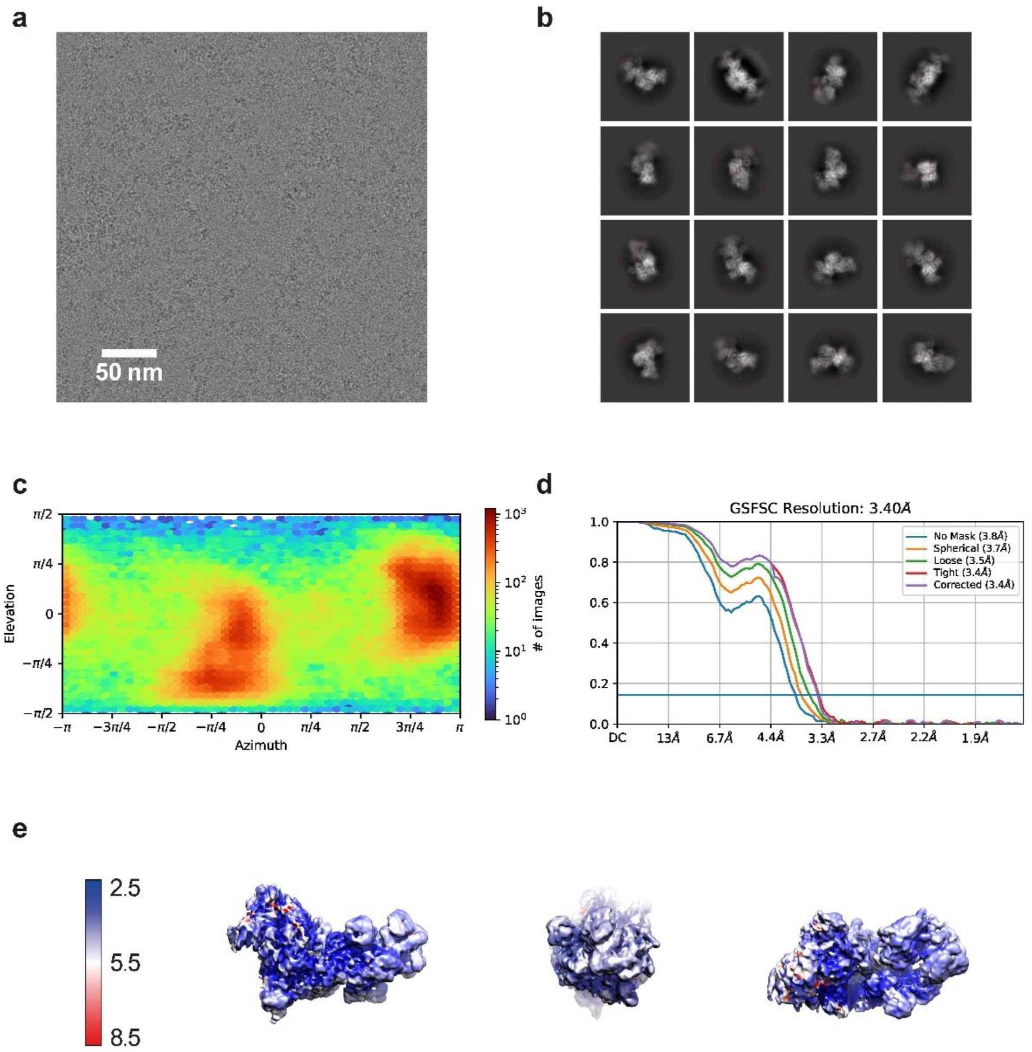

Supplementary Figure 2: CryoEM/SPA analysis of an ASCC3^{HR}-TRIP4 complex.

a, Representative cryoEM micrograph of the ASCC3^{HR}-TRIP4 complex. Scale bar, 50 nm. 6,266 micrographs were recorded from the same sample over two sessions, particle images were picked from 6,022 high-quality micrographs. The experiment was not repeated independently. **b**, 2D class averages of ASCC3^{HR}-TRIP4 particle images after reference-free 2D classification. **c**, Viewing direction distribution plot of the particle images used for the final reconstruction of the ASCC3^{HR}-TRIP4 cryoEM map as obtained during NU refinement with cryoSPARC. **d**, Global resolution estimation for the ASCC3^{HR}-TRIP4 cryoEM reconstruction by gold-standard Fourier shell correlation (FSC). Blue line, FSC$_{0.143}$. **e**, Local resolution estimation as determined with cryoSPARC, ranging from 2.5 Å to 35 Å for the ASCC3^{HR}-TRIP4 cryoEM reconstruction.

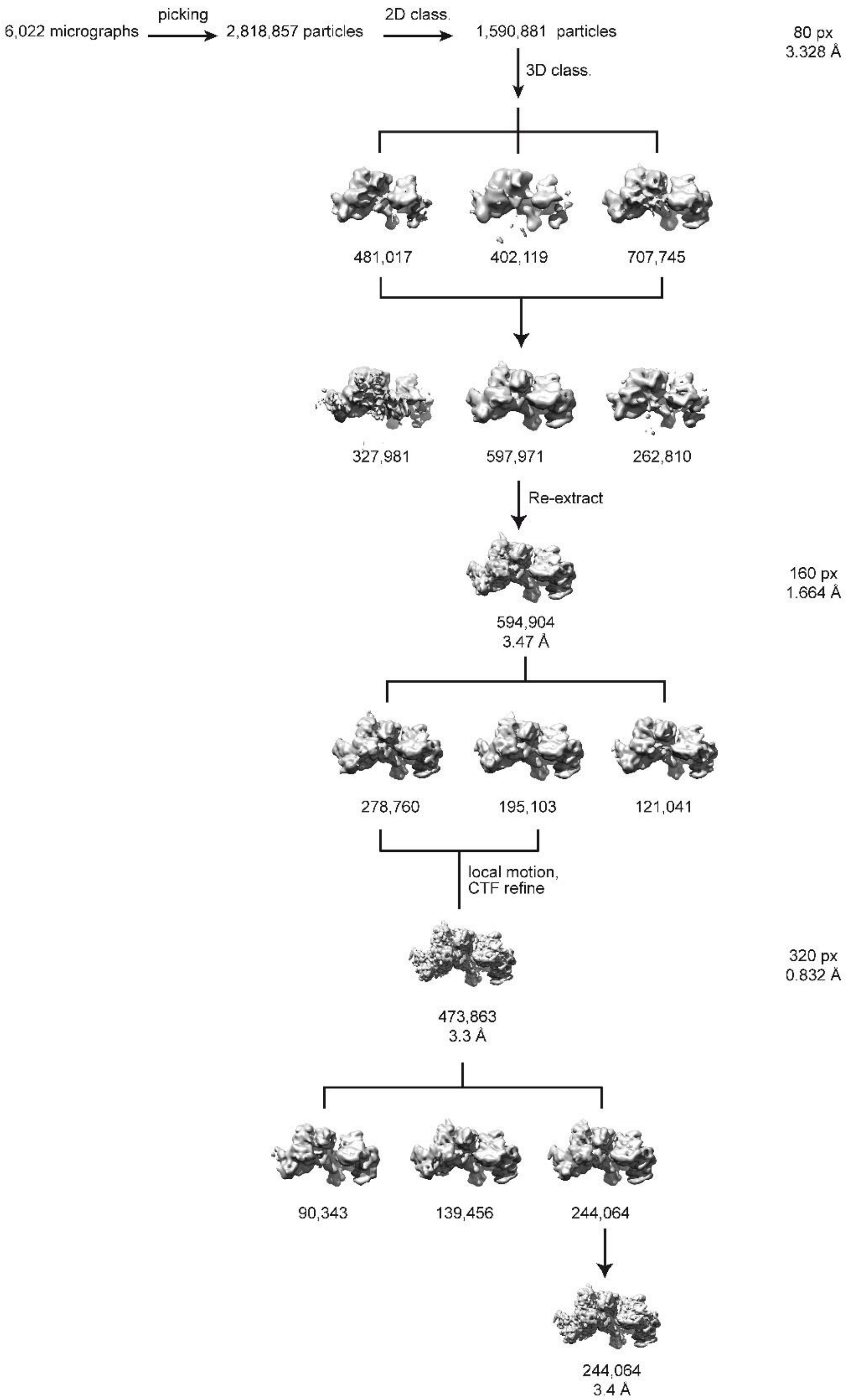

Supplementary Figure 3: CryoEM data refinement.

Reconstruction of the ASCC3^{HR}-TRIP4 cryoEM map. 2,818,857 particle images were picked from 6,022 micrographs and subjected to reference-free 2D classification. 1,590,881 particle images were selected for iterative cycles of heterogeneous 3D refinement into 3 classes. The

best appearing class, consisting of 597,971 particle images, was selected for re-extraction with a box size of 160 px (1.664 Å/px). Homogeneous NU refinement yielded a reconstruction at 3.47 Å resolution. Another heterogeneous refinement was done after which 473,863 particle images were selected for local motion correction with re-extraction at full spatial resolution with a box size of 320 px (0.832 Å/px). NU refinement followed by CTF refinement yielded a reconstruction at 3.3 Å resolution. A last iteration of heterogeneous refinement was applied to select 244,064 particle images were for final NU refinement yielding a reconstruction at 3.4 Å resolution.

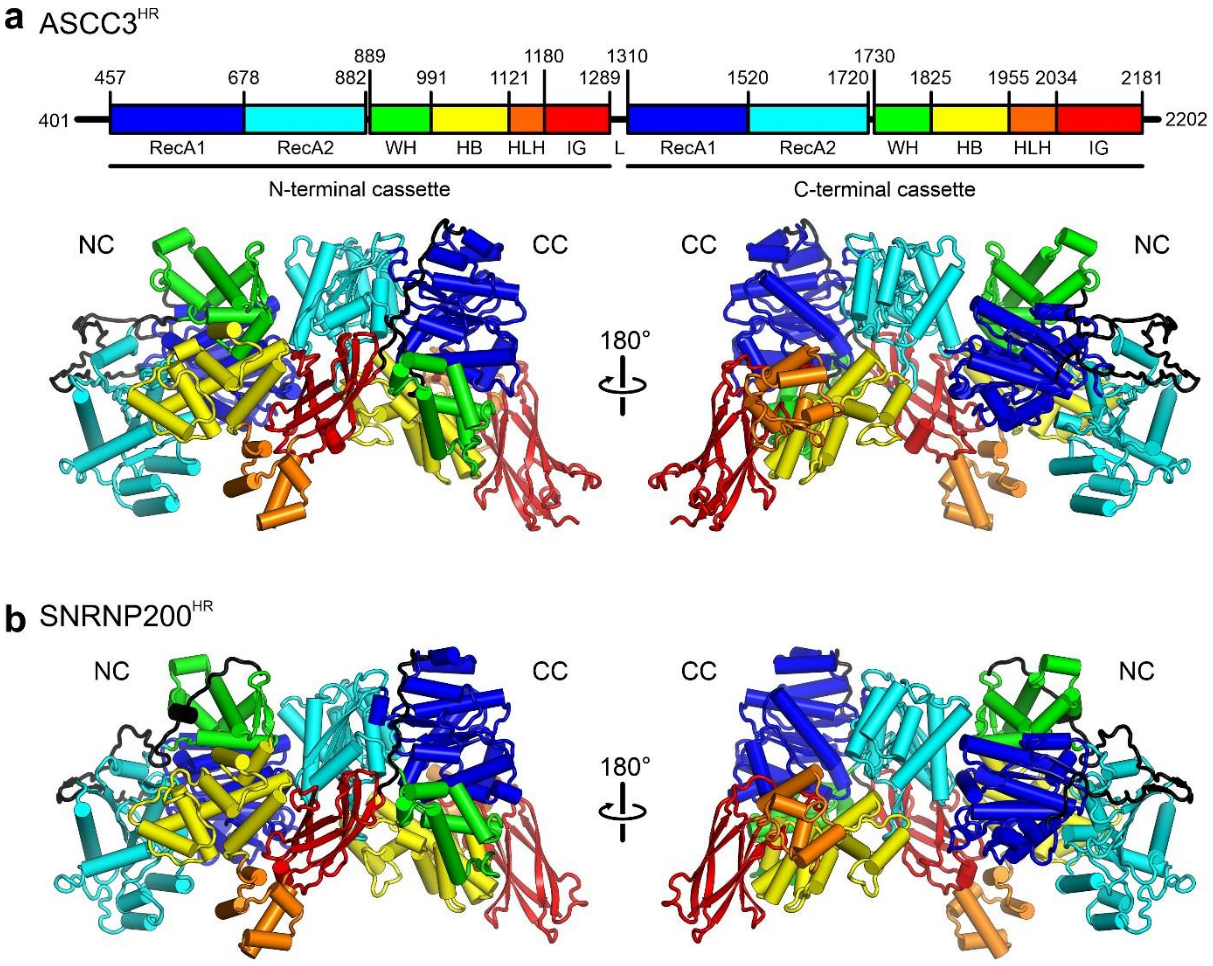

Supplementary Figure 4: Global comparison of ASCC3^{HR} to SNRNP200^{HR}.

a, Domain scheme (top) and cartoon representation (bottom; 180° views) of ASCC3^{HR}. Numbers above the scheme represent domain borders. RecA1/2, RecA-like domains; WH, winged-helix domains; HB, helical bundle domains; HLH, helix-loop-helix domains; IG, immunoglobulin-like domains. Domains are colored blue to red from N-terminus to C-terminus within each helicase cassette. Orientation of the left panel as in Fig. 1c,d, left. **b**, Cartoon representation of SNRNP200^{HR} (PDB ID 4F91)[11] after global alignment with ASCC3^{HR} in the same orientations and with equivalent domain coloring.

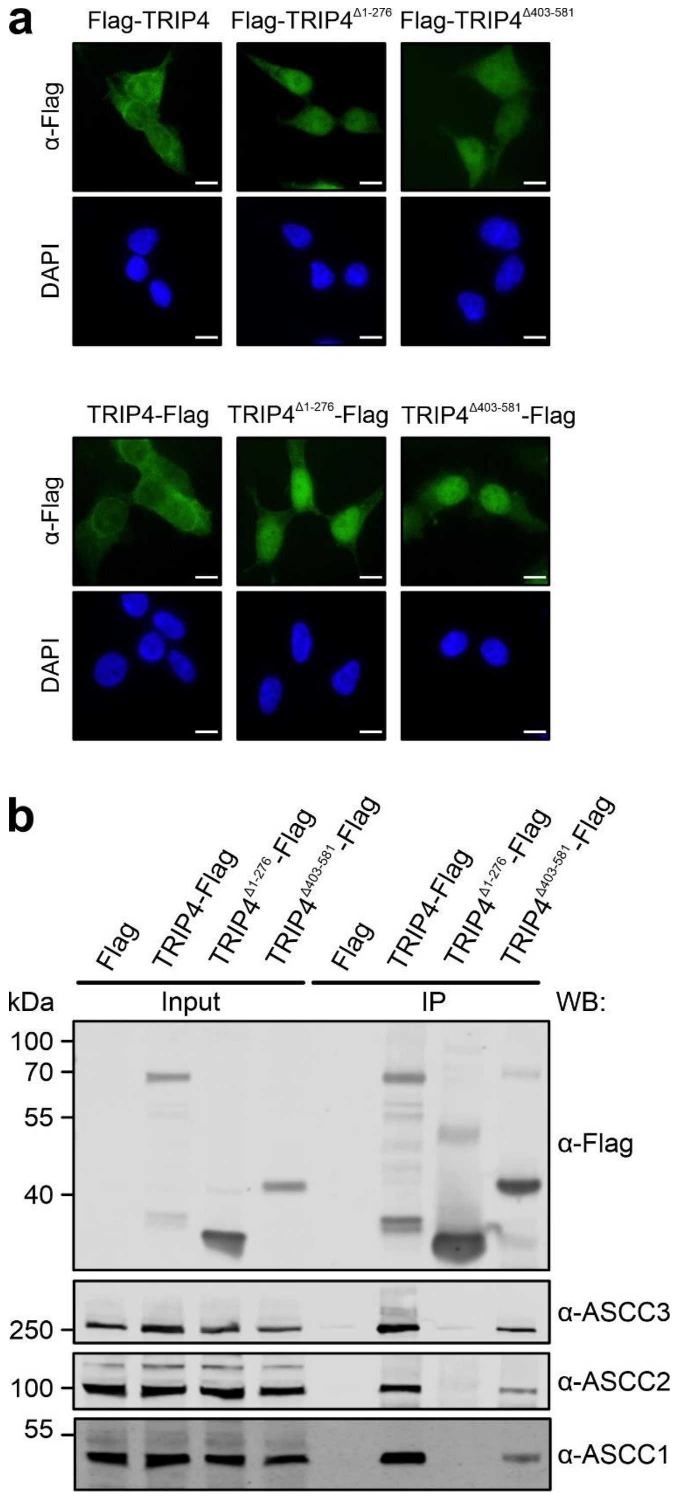

Supplementary Figure 5: Localization and interactions of TRIP4 variants in cells.

a, Immuno-fluorescence microscopy of Flp-In™ T-REx™ 293 cells stably expressing Flag-tagged TRIP4 variants (identified on the top; N-terminal Flag-tag, top panels; C-terminal Flag-tag, bottom panels) after staining with α-Flag antibody (top rows) and DAPI (bottom rows), revealing nuclear and cytosolic localization of all TRIP4 variants. Scale bars, 10 µm. **b**,

Western blots (WB) monitoring immuno-precipitation (IP) of ASCC1, ASCC2 and ASCC3 by the indicated C-terminally Flag-tagged TRIP4 variants from cell extracts. Experiments were repeated independently three times with similar results. Source data are provided as a Source Data file.

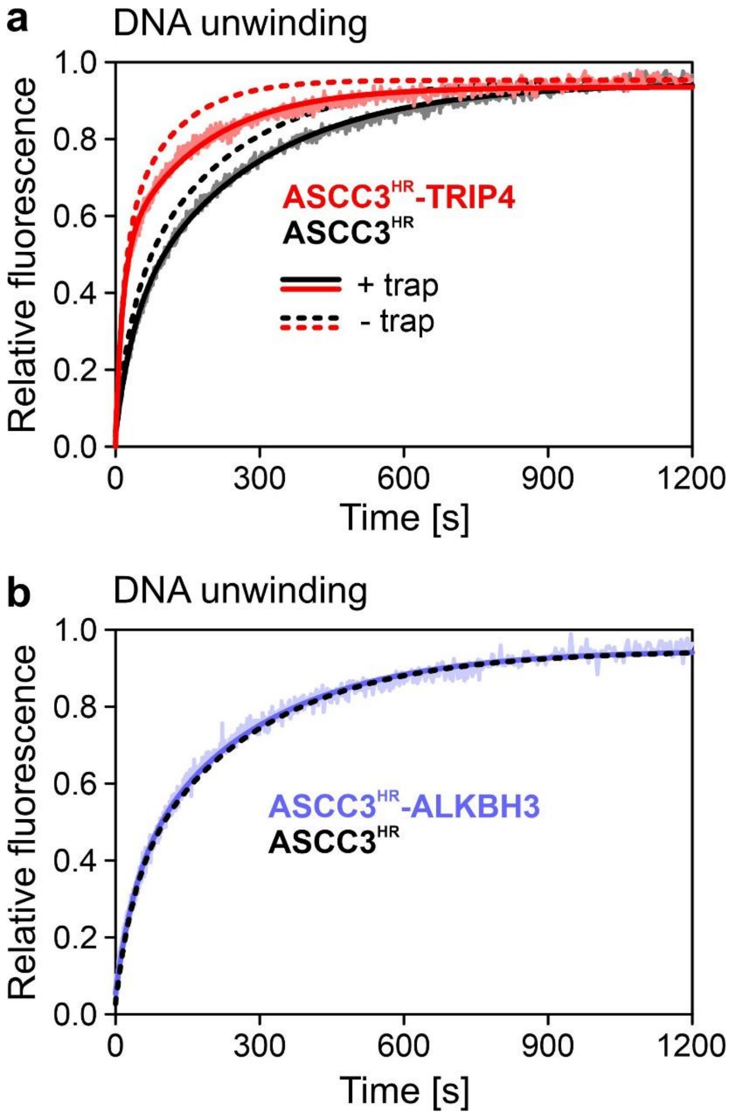

Supplementary Figure 6: Stopped-flow/fluorescence-based DNA unwinding in presence of a DNA trap.

a, Stopped-flow/fluorescence-based DNA unwinding by ASCC3^{HR} or ASCC3^{HR}-TRIP4 in the presence of a DNA trap (data and solid curve fits), revealing similar time traces as in the absence of a trap. Dashed lines, curve fits for DNA unwinding by ASCC3^{HR} or ASCC3^{HR}-TRIP4 in the absence of a DNA trap from Fig. 3a. **b**, Stopped-flow/fluorescence-based DNA unwinding by ASCC3^{HR}-ALKBH3 in the presence of a DNA trap (data and solid curve fit), revealing lack of stimulation of the of the by ASCC3^{HR} helicase by ALKBH3. Dashed line, curve fit for DNA unwinding by ASCC3^{HR} in the presence of a DNA trap from panel (**a**). Source data are provided as a Source Data file.

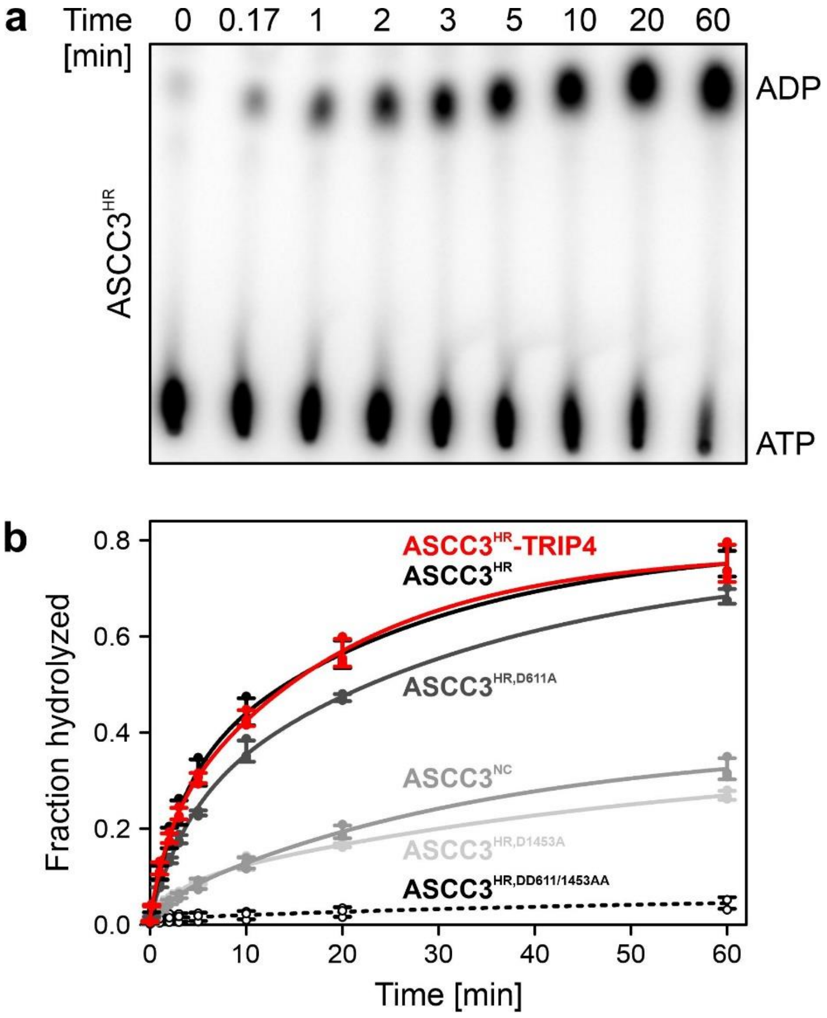

Supplementary Figure 7: DNA-stimulated ATPase activities.

a, Exemplary thin-layer chromatogram showing a time course of DNA-stimulated ATP hydrolysis by ASCC3^{HR}. **b**, Quantification of data as shown on (**a**). Individual data points (spheres) with means ± SD (lines) for n = 3 technical replicates are shown. Curves show fits of the data to $V = (A_{fast} * V_{fast}^2 + A_{slow} * V_{slow}^2) / (A_{fast} * V_{fast} + A_{slow} * V_{slow})$; $A_{fast/slow}$, amplitudes of the fast/slow hydrolysis phases; $V_{fast/slow}$, rates of the fast/slow hydrolysis phases [min^{-1}]; V, ATP hydrolyzed as a function of time [min^{-1}]. Source data are provided as a Source Data file.
