# Supplementary material for: Extended DNA threading through a dual-engine motor module of the activating signal co-integrator 1 complex
Source: Nat Commun. 2023 Apr 5;14:1886. doi: 10.1038/s41467-023-37528-3 (PMC10076317; doi:10.1038/s41467-023-37528-3)
Supplement: Supplementary file 3 — Reporting Summary [file 41467_2023_37528_MOESM3_ESM.pdf]

Corresponding author(s): Markus C. Wahl
Last updated by author(s): Jan 24, 2023

Reporting Summary

Nature Portfolio wishes to improve the reproducibility of the work that we publish. This form provides structure for consistency and transparency in reporting. For further information on Nature Portfolio policies, see our Editorial Policies and the Editorial Policy Checklist.

Statistics

For all statistical analyses, confirm that the following items are present in the figure legend, table legend, main text, or Methods section.

n/a	Confirmed	
☐	☒	The exact sample size (*n*) for each experimental group/condition, given as a discrete number and unit of measurement
☐	☒	A statement on whether measurements were taken from distinct samples or whether the same sample was measured repeatedly
☐	☒	The statistical test(s) used AND whether they are one- or two-sided<br>*Only common tests should be described solely by name; describe more complex techniques in the Methods section.*
☒	☐	A description of all covariates tested
☒	☐	A description of any assumptions or corrections, such as tests of normality and adjustment for multiple comparisons
☐	☒	A full description of the statistical parameters including central tendency (e.g. means) or other basic estimates (e.g. regression coefficient) AND variation (e.g. standard deviation) or associated estimates of uncertainty (e.g. confidence intervals)
☐	☒	For null hypothesis testing, the test statistic (e.g. *F*, *t*, *r*) with confidence intervals, effect sizes, degrees of freedom and *P* value noted<br>*Give P values as exact values whenever suitable.*
☒	☐	For Bayesian analysis, information on the choice of priors and Markov chain Monte Carlo settings
☒	☐	For hierarchical and complex designs, identification of the appropriate level for tests and full reporting of outcomes
☒	☐	Estimates of effect sizes (e.g. Cohen's *d*, Pearson's *r*), indicating how they were calculated

Our web collection on statistics for biologists contains articles on many of the points above.

Software and code

Policy information about availability of computer code

Data collection	EPU, version 2.14 (cryoEM/SPA data acquisition; Thermo Fisher Scientific; referenced in text)
Data analysis	ChimeraX, version 1.4 (structure figure preparation; referenced in text)<br>Coot, version 0.9.8.1 (model building; referenced in text)<br>cryoSPARC, version 3.2.2 (all cryoEM image analysis; referenced in text)<br>ImageQuant, version 5.2 (quantification of bands on gels; Cytiva; referenced in text)<br>Molprobity, version 4.5.1 (structure evaluation; referenced in text)<br>OpenNuXL node of OpenMS, version 3.0.0 (cross-link data analysis; referenced in text)<br>PHENIX, version 1.20_4459 (real space refinement; referenced in text)<br>PISA sever, version 1.52 (macromolecular interface analysis; referenced in text)<br>PyMOL, version 1.8 (rmsd calculation; structure figure preparation; Schrödinger, LLC; referenced in text)<br>Prism, version 9.0 (data fitting; GraphPad; referenced in text)

For manuscripts utilizing custom algorithms or software that are central to the research but not yet described in published literature, software must be made available to editors and reviewers. We strongly encourage code deposition in a community repository (e.g. GitHub). See the Nature Portfolio guidelines for submitting code & software for further information.

Data

Policy information about availability of data

All manuscripts must include a data availability statement. This statement should provide the following information, where applicable:

- Accession codes, unique identifiers, or web links for publicly available datasets
- A description of any restrictions on data availability
- For clinical datasets or third party data, please ensure that the statement adheres to our policy

The cryoEM reconstruction of the ASCC3HR-TRIP4 complex has been deposited in the Electron Microscopy Data Bank (https://www.ebi.ac.uk/pdbe/emdb) under accession code EMD-15521 (https://www.ebi.ac.uk/pdbe/entry/emdb/EMD-15521). Structure coordinates have been deposited in the RCSB Protein Data Bank (https://www.rcsb.org) with accession code 8ALZ (https://www.rcsb.org/structure/8ALZ).60 The DNA-protein CLMS data have been deposited in the ProteomeXchange Consortium (http://www.proteomexchange.org) via the PRIDE61 partner repository (https://www.ebi.ac.uk/pride/) under dataset identifier PXD036106 (https://www.ebi.ac.uk/pride/archive/projects/PXD036106). All other data are contained in the manuscript or the Supplementary Information. Source data are provided with this paper. Structure coordinates used in this study are available from the RCSB Protein Data Bank (https://www.rcsb.org) under accession codes 2P6R (https://www.rcsb.org/structure/2P6R) and 4F91 (https://www.rcsb.org/structure/4F91).

Human research participants

Policy information about studies involving human research participants and Sex and Gender in Research.

Reporting on sex and gender	N/A
Population characteristics	N/A
Recruitment	N/A
Ethics oversight	N/A

Note that full information on the approval of the study protocol must also be provided in the manuscript.

Field-specific reporting

Please select the one below that is the best fit for your research. If you are not sure, read the appropriate sections before making your selection.

☒ Life sciences ☐ Behavioural & social sciences ☐ Ecological, evolutionary & environmental sciences

For a reference copy of the document with all sections, see nature.com/documents/nr-reporting-summary-flat.pdf

Life sciences study design

All studies must disclose on these points even when the disclosure is negative.

Sample size	Sample sizes are described in the Methods. The sample sizes are standard for the in vitro assays performed in the study.<br>For cryoEM analysis, the sample size was chosen so as to yield a large number of particle images on the grids while avoiding non-specific aggregation.<br>For biochemical and biophysical assays, sample sizes were chosen so as to provide significant and reproducible signals (analytical SEC; unwinding assay, ATPase assay) or to provide clearly visible and quantifiable bands on gels (SDS PAGE analyses of analytical SEC).<br>For DNA-protein cross-linking/MS analyses, samples sizes were chosen so as to obtain clear signals in MS.
Data exclusions	Structural analysis by cryoEM involved the sorting of high quality particle images and rejection of poor quality particle images. Poor quality particle images may be due to compositional heterogeneity in the sample or may originate from particles being damaged in the process of grid preparation, e.g. at the air-water interface. The processing steps involving data exclusion are outlined in Supplementary Figure 2.<br>For other experiments, no data were excluded from the analyses.
Replication	The numbers of technical and biological replicates are indicated in the text and figure legends.<br>For interaction tests, unwinding assays and ATPase assays at least two independent experiments were performed using the same biochemical samples (such as recombinant proteins, nucleic acids, cell lines, antibodies). All attempts at replication were successful.
Randomization	This study reports results from in vitro biochemical/biophysical experiments. Randomization was not required for this study, as (1) no human or animal subjects were studied, (2) quantitative data were collected, (3) no subjective interpretations were required and (4) there was no danger of confounding independent variables in the experimental design.
Blinding	This study reports results from in vitro biochemical/biophysical experiments, for which blinding is not applicable, as the experiments did not involve human or animal subjects, and as the results from the experiments can be objectively evaluated/quantified.

Reporting for specific materials, systems and methods

We require information from authors about some types of materials, experimental systems and methods used in many studies. Here, indicate whether each material, system or method listed is relevant to your study. If you are not sure if a list item applies to your research, read the appropriate section before selecting a response.

Materials & experimental systems

n/a	Involved in the study
☐	☒ Antibodies
☐	☒ Eukaryotic cell lines
☒	☐ Palaeontology and archaeology
☒	☐ Animals and other organisms
☒	☐ Clinical data
☒	☐ Dual use research of concern

Methods

n/a	Involved in the study
☒	☐ ChIP-seq
☒	☐ Flow cytometry
☒	☐ MRI-based neuroimaging

Antibodies

Antibodies used

Monoclonal α-Flag-M2 antibody produced in mouse, clone M2, purified IgG1 subclass (Sigma-Aldrich F3165), diluted 1:7500
Monoclonal α-Flag-M2-FITC antibody produced in mouse, clone M2 (Sigma-Aldrich F4049), diluted 1:200
Monoclonal α-TRIP4 antibody (F-7), mouse/IgG1 κ (Santa Cruz sc-376916), diluted 1:1000
Polyclonal α-ASCC1 antibody, rabbit/IgG (Proteintech 12301-1-AP), diluted 1:500
Polyclonal α-ASCC2 antibody, rabbit/IgG (Proteintech 11529-1-AP), diluted 1:1000
Polyclonal α-ASCC3 antibody, rabbit/IgG (Proteintech 17627-1-AP), diluted 1:1000
Recombinant α-HA-tag antibody, rabbit monoclonal [EPR22819-101] to HA-tag (Abcam ab236632), diluted 1:4000

Validation

Monoclonal α-Flag-M2 antibody produced in mouse, clone M2, purified IgG1 subclass (Sigma-Aldrich F3165): https://www.sigmaaldrich.com/US/en/product/sigma/f3165
Monoclonal α-Flag-M2-FITC antibody produced in mouse, clone M2 (Sigma-Aldrich F4049): https://www.sigmaaldrich.com/US/en/product/sigma/f4049
Monoclonal α-TRIP4 antibody (F-7), mouse/IgG1 κ (Santa Cruz sc-376916): https://www.scbt.com/p/asc-1-antibody-f-7?requestFrom=search
Polyclonal α-ASCC1 antibody, rabbit/IgG (Proteintech 12301-1-AP): https://www.ptglab.com/products/ASCC1-Antibody-12301-1-AP.htm
Polyclonal α-ASCC2 antibody, rabbit/IgG (Proteintech 11529-1-AP): https://www.ptglab.com/products/ASCC2-Antibody-11529-1-AP.htm
Polyclonal α-ASCC3 antibody, rabbit/IgG (Proteintech 17627-1-AP): https://www.ptglab.com/products/ASCC3-Antibody-17627-1-AP.htm
Recombinant α-HA-tag antibody, rabbit monoclonal [EPR22819-101] to HA-tag (Abcam ab236632): https://www.abcam.com/ha-tag-antibody-epr22819-101-ab236632.html

Eukaryotic cell lines

Policy information about cell lines and Sex and Gender in Research

Cell line source(s)

Flp-In™ T-REx™ 293 cells (Thermo Fisher Scientific R78007)
Lenti-X 293T cells (Takara Bio 632180)
293T cells (ATCC CRL-3216)
PC-3 cells (ATCC CRL-1435)
Sf9 cells (Thermo Fisher Scientific 11496015)
High Five cells (Thermo Fisher Scientific B85502)

Authentication

Flp-In™ T-REx™ 293 cells were purchased from the company and were not further authenticated.
293T and PC-3 cells were purchased from the company and were authenticated using the ATCC human STR profiling services.
Parental Sf9 cells and High Five cells were purchased from the company and were not further authenticated. Baculovirus-based production of target proteins was confirmed by detecting target proteins on SDS PAGE gels after affinity purification.
Lenti-X 293T, 293T, PC-3, Sf9 and High Five cells are lab stocks that have been extensively used.

Mycoplasma contamination

Flp-In™ T-REx™ 293, LentiX-293T, 293T and PC-3 cells were tested for mycoplasma contamination and confirmed to be negative.
Sf9 cells and High Five cells were not tested for for mycoplasma contamination, as cells were only used for recombinant protein production.

Commonly misidentified lines
(See ICLAC register)

No commonly misidentified cell lines were used in the study.
